# [H1] The widened gap in mental health services during the pandemic

**DOI:** 10.1016/j.lanwpc.2021.100320

**Published:** 2021-10-29

**Authors:** The Lancet Regional Health – Western Pacific

WHO defines health as “a state of complete physical, mental and social wellbeing and not merely the absence of disease or infirmity”. In the pursuit of physical health, people have never hesitated to combat disease. By contrast, our recognition of the existence of mental disorders and the essential need to take care of mental health has seen a long delay. Nowadays, with the notion of “no health without mental health”, both individual and societal recognition of mental health have vastly improved. Individuals are in transition from recognising the issue and accepting the need for help, to developing the intention and seeking the required help. On a societal level, efforts are being made to spread knowledge, reduce stigma, and develop mental health services in prevention, diagnosis, and intervention. However, these efforts are far from enough when we look at the substantial gap between the demand for mental health services and supply, which has widened during the COVID-19 pandemic.

Globally, government health budgets included an average of only 2% for mental health in recent years. Development assistance for mental health has never exceeded 1% of the total development assistance for health. In low-income and middle-income countries (LMICs), the number is only 0•5%. Mental health care is under-resourced and receives the least investment compared with other health conditions in many countries. In the Western Pacific region, more than 100 million people have mental health disorders, while the median number of mental health workers in the region is ten per 100 000 population. Worryingly, 76–85% of people with severe mental disorders receive no treatment in LMICs in the region. These already large treatment gaps in mental health care are expected to have worsened during the COVID-19 pandemic, attributed to the increased burden of mental disorders, and disrupted mental health services.

On Oct 8, 2021, The Lancet published global estimates of the impact of the pandemic on mental health. An estimated 53•2 million additional cases of major depressive disorder (an increase of 27•6%) and 76•2 million additional cases of anxiety disorders (an increase of 25•6%) were diagnosed during the COVID-19 pandemic. Locations hit hardest by the pandemic in 2020 had the highest increases in mental health diagnoses.

Even after the pandemic, uncertainties about sporadic outbreaks of the disease, emergence of new variants, effectiveness of vaccines, and when and to what extent everyday life will return to normal are constantly imposing stressors leading to higher risks of mental disorders, including anxiety and depression.

Unfortunately, at a time when they are greatly needed, mental health services have been disrupted, further exacerbating the gap between demand for mental health services and supply.

Before the pandemic, mental health screening, prevention, and intervention services and programmes were mainly set up in schools, workplaces, and within communities. However, due to the implementation of strict measures to contain the pandemic, including lockdowns, school closures, and travel restrictions, large numbers of these services were disrupted, resulting in inaccessible mental health care services for people in need. According to a WHO report released on Oct 5, 2020, three-quarters of school or workplace mental health services were wholly or partly disrupted due to COVID-19, as well as community-based services, which are already limited in availability.

Fortunately, some actions are being taken to respond to the widened gap in demand and supply for mental health services. Innovative service methods have been developed to overcome the disruption to existing services. Digital interventions, such as hotlines for remote psychological first aid and apps allowing access to mental health specialists have been introduced; materials and guidelines have been developed and delivered via social media to encourage self-help and help-seeking among susceptible groups; and training in basic psychosocial skills for health-care providers, counsellors, and social workers have been rolled out.

Now more than 18 months after the outbreak of the COVID-19 pandemic, mental health, as a far-reaching result of the pandemic has been brought to the forefront. As the theme of this year's World Mental Health Day, it is time to make mental health care for all a reality by addressing the gap in mental health services during the pandemic.

